# Evidence-based classification of low back pain in the general population: one-year data collected with SMS Track

**DOI:** 10.1186/2045-709X-21-30

**Published:** 2013-09-02

**Authors:** Charlotte Leboeuf-Yde, Nadège Lemeunier, Niels Wedderkopp, Per Kjaer

**Affiliations:** 1 Research Department, The Spine Center of Southern Denmark, Hospital Lillebælt and Institute of Regional Health Research, University of Southern Denmark, Østre Hougvej 55, DK5500 Middelfart, Denmark; 2 Complexité, Innovation et Activités Motrices et Sportives, Bâtiment 335, UFR STAPS, Université Paris Sud-11, Orsay Cedex 91405, France; 3 Institut Franco-Européen de Chiropraxie, 72 Chemin de la Flambère, 31300 Toulouse, France; 4 Orthopaedic Department, Center for Spine Surgery, Hospital of Lillebaelt, Institute of Regional Health Service Research and Center for Research in Childhood Health, University of Southern Denmark, Østre Hougvej 55, DK5500 Middelfart, Denmark; 5 Department of Sports Science and Clinical Biomechanics, University of Southern Denmark, Campusvej 55, DK5230 Odense M, Denmark

**Keywords:** Low back pain, Epidemiology, Trajectories, Subgroups, Episodes, Text-messages

## Abstract

**Background:**

It was previously assumed that low back pain (LBP) is a disorder that can be classified as acute, subacute and chronic. Lately, the opinion seems to have veered towards a concept of it being a more recurrent or cyclic condition. Interestingly, a recent review of the literature indicated that LBP in the general population is a rather stable condition, characterized as either being present or absent. However, only one of the reviewed studies had used frequent data collection, which would be necessary when studying detailed course patterns over time. It was the purpose of this study to see, if it was possible to identify whether LBP, when present, is rather episodic or chronic/persistent. Further, we wanted to see if it was possible to describe any specific course profiles of LBP in the general population.

**Methods:**

In all, 293 49/50-yr old Danes, who previously participated in a population-based study on LBP were invited to respond to 26 fortnightly text-messages over one year, each time asking them the number of days they had been bothered by LBP in the past two weeks. The course patterns for these individuals were identified through manual analysis, by observing the interplay between non-episodes and episodes of LBP. A *non*-*episode of LBP* was defined as a period of at least one month without LBP as proposed by de Vet et al. A fortnight with at least one day of pain was defined as a *pain fortnight* (*FN*). At least one pain FN surrounded by a non-episode on each side was defined as an *episode of LBP*. After some preliminary observations of the spread of data, episodes were further classified as *brief* (consisting of only one pain FN) or *longer* (if there were at least 2 pain FNs in a row). An episode of at least 6 pain FNs in a row (i.e. 3 months) was defined as a *long*-*lasting episode*.

**Results:**

In all, 261 study subjects were included in the analyses, for which 7 distinct LBP subsets could be identified. These could be grouped into three major clusters; those mainly without LBP (35%), those with episodic LBP (30%) and those with persistent LBP (35%). There was a positive association between number of episodes and their duration.

**Conclusion:**

In this study population, consisting of 50-yr old persons from the general population, LBP, when present, could be classified as either ‘episodic’ or ‘mainly persistent’. About one third was mainly LBP-free throughout the year of study. More information is needed in relation to their relative proportions in various populations and the clinical relevance of these subgroups.

## Background

Almost 45 years ago, in 1969, an orthopaedic surgeon, LM Rowe, [1] suggested that backache is a “characteristically intermittent, episodic and recurrent” disorder that should be “studied only as a continuum”. However, low back pain (LBP) did not seem to evoke much interest again until about twenty years later and, at that time, it was generally thought to be a benign condition, usually with a good prognosis. The majority of people with LBP (80-90%) were thought to have recovered within 6 weeks, at least in terms of return to work [2-4], although that outcome seems often to have been interpreted to relate also to the pain experience. Therefore, LBP was [5] and, in fact, still often is [6,7] defined as either acute or chronic.

Epidemiologic studies on LBP in the general population have typically consisted of cross-sectional surveys, either single or several in a row. In such studies, annual rates of incidence (i.e. incidence of first-time LBP) have been identified and reported to range between 1% and 16%, and even (an unrealistic) 94% [8]. This would mean that in the end, everybody would have experienced LBP. In other words, the concept of LBP was that of a disorder that could start at any time in life to disappear rapidly for most, but when it did not disappear quickly, it turned into a chronic condition, i.e. it was there to last forever. The cut-point for the onset of ‘chronic’ has been set in various ways, but one frequently used definition is three months.

Little by little, the view on LBP changed, again to be seen as more of an episodic, cyclic disorder [9] and some even bet on both horses, i.e. considering it to be both a short-lasting and a recurrent problem [10], as the one does not exclude the other.

No generally accepted classification or description of these episodes and cycles seem to exist, although it has been proposed that an ‘episode’ should be defined as two non-episodes surrounding at least one day with 24 hours of symptoms [11]. A ’non-episode’ has been suggested to consist of four consecutive weeks without LBP [11]. It is not clear what requirements there are for the number, duration and frequency of such episodes, for LBP to be diagnosed as ‘episodic’ or ‘recurrent’. Also, we found no generally accepted definitions of the spacing of such episodes, nor for the duration between the two extreme points, required for the definition of recurrent, episodic LBP.

In fact, according to a review, a wide variety of definitions of recurrency has been found in the literature [12] and despite a recent attempt at reaching agreement [13], no evidence-based definition exists of episodic LBP.

On a different tangent, a recent literature review of course-studies in the general population revealed quite clearly that regardless definition of LBP, and the time and number of follow-ups, LBP is a fairly stable condition, particularly among those who do *not* have it [14]. When changes occur over time, such changes will only be modest. Rarely was it shown to go from no pain to frequent or persistent pain, or changing from frequent/persistent pain to no pain. This finding is in contradiction with the benign/good prognosis theory and possibly also with the episodic theory, instead indicating that LBP should be characterized as a rather stable condition in the general population.

However, only one of the studies that formed the basis for this conclusion had used frequent data collection [15], which would be necessary when studying detailed course patterns over time. No doubt, the access to new technical tools will change the methods of data collection in back research. One such novel technique, frequent data collection through text messaging (SMS-Track), was used in a study of the general population. This made it possible to see whether there are some major subgroups that fit with the recent findings and theories. Specifically, we wanted to see if, over a period of one year, we could identify 1) a stable LBP free subgroup, 2) a subgroup with predominantly episodic LBP, and 3) a subgroup with a more persistent LBP profile. Further, we were interested in any specific profiles of absence and presence of symptoms among those with LBP.

## Method

### Study subjects, data collection, variables of interest, and ethics approval

Invited for the present study were those 293 individuals, who remained in a Danish population-based study ten years after the initial survey in 2000, and who accepted participation in the present study. They all turned 50 during the year of the study. Participants received identical automated text messages every fortnight over one year (26 times), using the SMS-Track-Questionnaire system [16]. They received two standardized questions relating to their low back in the past fortnight; one on the number of days that their lower back had bothered (troubled) them and the other on the number of days on sick-leave in the past fortnight. In the present study, only data on LBP were used. Their fortnightly response, which was sent as a return text message to the SMS-Track question, was in relation to the number of days in this period with bothersome LBP, i.e. they responded with a number from 0 to 14.

We used de Vet et al’s. definition of a non-episode (one month) and their recommendation that an episode had to consist of at least one day of LBP [11]. Unfortunately, we could not adhere to their exact definition of new pain, which is in fact, 24 hours (hrs) of pain, as we had no information on the duration of pain in our study subjects. However, we consider this definition of 24 hrs of pain as somewhat misleading. It would probably be unusual that people with LBP have constant pain for at least 24 hrs. Experience indicates that their pain would depend on positions, movements and time of the day, thus not a constant presence for that entire period. Nevertheless, we appreciate that the ’24 hrs of pain’-definition has been used in order to exclude the occasional twinge or ache. The fact that we used “bothered by LBP” as our definition of LBP makes it likely to obtain positive answers from people who suffered LBP of some personal consequence [17] and not from those with the occasional twinge or ache, and we therefore assume that our data would meet the expectations of de Vet et al.

The initial epidemiologic study, including a description of representativeness of the study sample, has been reported in detail elsewhere [18]. A summary of base-line data is provided in Table 1. Ethics and data management approvals were obtained from the relevant authorities (Ethics Committee ref number 200000042; Danish Data Protection Agency ref number 2000-53-0037).

**Table 1:** **Baseline description of the study subjects consisting of 50-yr olds from the general Danish population who participated in the baseline study** (**N** = **412**) **and those participating in the present SMS**-**Track survey** (**N** = **261**)

**Variables of interest**	**First survey 10 yrs prior to present study**	**Present SMS-Track survey**
	**N (%)**	**N (%)**
Proportion women	213 (52)	142 (54)
Employment status		
Self employed	29 (7)	18 (7)
Assisting spouse	2 (0)	0 (0)
Employed	344 (84)	230 (88)
Unemployed	17 (4)	7 (3)
Pensioner	11 (3)	2 (1)
Others outside labor force	9 (2)	4 (2)
Highest educational level		
Basic school	92 (22)	49 (19)
General upper-secondary education	9 (2)	6 (2)
Vocational education/training	127 (31)	86 (33)
Short-cycle higher education	84 (20)	56 (21)
Medium-cycle higher education	77 (19)	50 (19)
Long-cycle higher education	23 (6)	15 (5)
Reported having had low back pain past year	284 (69)	170 (65)
Number of days with low back pain the year preceding study		
0 days	123 (30)	86 (33)
1–30 days	187 (45)	121 (46)
>30 days	102 (25)	54 (21)
Reported having been on sick-leave preceding year because of low back pain		
0 days	329 (80)	201 (77)
1–30 days	61 (15)	51 (20)
>30 days	22 (5)	9 (3)

Information obtained from a Danish general population of 50-yr olds, who in the last survey were followed with fortnightly text-messages over one year.

### Quality of data, data management and analysis of data

The text message replies went straight into a data file, which could be accessed by the researchers at any time. The experience of researchers who have used this method is that it is possible to obtain good collaboration from the study subjects, but success requires that clear information is given at the onset of the study. It is also necessary to supervise the input of answers, particularly at the beginning, which can be done by accessing the spread-sheet where each respondent’s response status can be viewed at any time. A research secretary was responsible for this task, and each fortnight she would call non-responders and people who answered incorrectly (e.g. in words instead of a number) to help them respond properly. This made it possible to obtain a high response rate and high quality data.

Those who failed to respond at least six of the 26 times were removed from the analysis. The occasional missing values were filled in through manual imputation. Imputation calculation was based on the total number of painful days reported divided by the number of returned messages. A *non*-*episode of LBP* was defined as a period of at least one month without LBP. A fortnight (FN) with at least one day of pain was defined as a *pain FN*. At least one pain FN surrounded by a non-episode on each side was defined as an *episode of LBP*. In order to get a feeling for the spread of data, a preliminary analysis was performed, where various patterns were grouped until it became clear that episodes could be classified as *brief* (consisting of only one pain FN) or *longer* (if there were at least 2 pain FNs in a row). An episode of at least 6 pain FNs in a row (i.e. 3 months) was defined (pre hoc) as a *long*-*lasting episode*, because this corresponds with ‘chronic’ as defined in numerous studies. The spread of data was thereafter identified for each individual using the above definitions. Specific patterns were thereafter searched out through visual analysis. The subgroups that emerged had therefore not been identified à priori but emerged after systematic observations.

Results have been reported as percentages with 95% confidence intervals. No further analyses were performed in relation to potential modifying factors or group characteristics. However, such analyses will be reported elsewhere.

## Results

### Response rate, and comparison between responders at baseline and in the present survey

Of the 293 invited, 16 declined and 16 failed to provide sufficient data, leaving 261 (89%) for the analyses. Imputation was performed on 2% of the answers (“cells”) (129/6708 cells) for 76 of the individuals. A comparison of the responders at the present survey with those at baseline shows the two study samples to be similar (Table 1).

### Number of subgroups

When the classification process had been completed, seven subsets had been identified. All subjects but 19 had been fitted into one of these groups. One of these was an error and could be moved into the appropriate category, whereas the remaining 18 were easily forced into the group to which they most closely resembled. Ten of these fitted into the category of having only one brief episode, but their brief episode occurred too early or too late in the course to have had a complete non-episode either before or after the reported pain FN.

### Description of subgroups

The final subgrouping revealed that 19% (95% CI: 14–24) reported no pain days at all and 12% (8–16) had pain more or less all the time, of which 7 individuals had pain every day of the year. For details of the spread of data, please refer to Table 2.

**Table 2:** **Seven low back pain** (**LBP**) **groups classified into three main clusters based on frequency and duration of episodes in a study sample from the general Danish population of 50-yr olds (N=261) followed fortnightly over 1 year**

**Main subgroups**	**Percentages of participants fitting into the main subgroups (95% confidence interval)**	**Subsets making up the main subgroups**	**Percentages of participants fitting into the subsets (95% confidence interval)**
More or less constant LBP	35% (29–41)	• LBP every day	3% (2.8-3.2)
		• No LBP free fortnights at all (but not pain every day)	9% (6–12)
		• At least one long-lasting period of LBP (i.e. at least 6 pain fortnights in a row) but none of the above.	23% (18–28)
Episodic LBP	30% (24–36)	• At least one longer episode of LBP (i.e. at least 2 pain fortnights in a row).	23% (18–28)
		• Several brief episodes of LBP (maximum of 1 pain fortnight and none longer than 1 fortnight.	6% (3–9)
More or less never LBP	35% (29–41)	• Only 1 brief episode of LBP (i.e. maximum of 1 pain fortnight) and no other short or longer episodes.	16% (12–20)
		• No LBP days at all.	19% (14–24)

Data obtained from a Danish general population of 50-yr olds (N = 261) followed fortnightly with text messages over one year. Percentages have been rounded off.

Most importantly, three major almost equally large groups emerged, namely: 1) those who mainly do not have LBP (35%; 29–41), 2) those who have it at times (30%; 24–36), and 3) those who have it more or less always (35%; 29–41) (Table 2).

Information on number of episodes and their duration is shown in Table 3. About half reported to have had 1–4 episodes, most commonly 1 or 2 episodes. About half also reported only short episodes; with the duration of the longest episode being only 1 pain FN. The number of episodes and their duration were linked together, with those who reported only 1 episode more often reporting a duration of only 1 FN. Conversely, the episodes tended to last longer for those who had more than 1 episode.

**Table 3:** **The fewer the number of episodes of low back pain** (**LBP**) **the shorter the duration of this episode and the larger the number of episodes the longer the duration of episodes in a study sample from the general Danish population of 50-yr olds (N=261) followed fortnightly over 1 year**

**Number of episodes with LBP per subject**	**Longest duration of episodes of LBP per subject was 1 pain fortnight**		**Longest duration of episodes of LBP per subject was** ≥ **2 pain fortnights**	
	**N**	**(%)**	**N**	**(%)**
1	39/57	(68)	18/57	(32)
2	21/41	(51)	20/41	(49)
3 or 4	13/37	(35)	24/37	(65)

Data obtained from a Danish general population of 50-year olds (N = 261) followed with fortnightly text-messages over one year.

## Discussion

### Summary of findings and some considerations

This appears to be the first study in which frequent data on the presence/absence of LBP in the general population were collected covering information for an entire year. The results of this study showed that SMS-Track data can be used successfully to classify people from the general population into three meaningful clinical subgroups; those without LBP, those with episodic LBP, and those with persistent LBP. These subgroups were approximately equally large in this particular study population.

The results support the findings from a recent literature review, showing that there is a substantial LBP-free subgroup in the general population and that, at least during one year, this group will remain LBP healthy. Our results also confirmed the episodic theory, in that such a subgroup was seen to definitely exist but, at least in this study sample, there was also an at least equally large chronic group, if chronic is defined as experiencing at least some pain each fortnight during a minimum period of 3 months.

This study revealed also that people, who only have the occasional experience of LBP, are unlikely to suffer for a long time. Clinically, this brings forth the picture of a person who hurt the back through some activity, and given a bit of time, the problem disappears, with or without treatment. On the other hand, the results show that the occurrence of several episodes is more likely to result in at least one longer episode of LBP, indicating perhaps a more serious problem or a different etiology.

A similar previous study of the general population [15] was conducted in 2010. Thus Tamcan et al. appear to have been the first team truly to have observed the detailed course of LBP in the general population, which was done over one year through a weekly pain dairy. Their study sample differed from ours in that LBP was an inclusion criterion. A stable profile was identified in 2/3 and a fluctuating course in the remaining 1/3. Their mean age (ca. 53) resembled our group (all 50) but they were spread between 18 and 75+. If chronicity is linked with age, this could perhaps explain why the authors of that study found more people with a stable course than we did. Since participation in their study depended on LBP at baseline, no comparison could be made on absence of symptoms.

Dunn et al. in [19] seem to have been the first group to have studied the detailed course pattern of LBP, using monthly data collection, also over one year. But since they included patients who consulted for LBP, their profile in relation to persistence (57%) and episodic (13%) is not comparable to ours. Nevertheless, both these studies indicate that LBP, when present, is rather persistent than episodic.

The previous two studies included information on severity of symptoms, which ours did not, making it impossible to compare our data to theirs in relation to this aspect.

### Methodological considerations

Positive aspects of our study are that it deals with a non-clinical study sample, as it is impossible to obtain information on the relative proportion of LBP and absence of LBP in patient populations. The study sample was originally randomly sampled from the general population, rather than obtained through adverts or in work places, and the response rate was high, which should result in a study sample that is fairly representative of the general population. Further, compliance was high each fortnight - missing data occurred in only 2% of cells - so it is unlikely that imputed data would have falsified the results to any larger degree. Further, the frequent data collection would combat memory decay, which would help produce valid data. The fact that all our respondents were of the same age is also an advantage, as it removed any possible modifying effects of age. On the other hand, this also limits the generalizability of our results to other age groups.

A potential limitation of this study is also that the final study sample did not consist of all the same people as those who were originally invited to participate in the study, because of the gradual drop-out rate. This is a common phenomenon seen in longitudinal studies with a number of follow-up surveys, and any possible bias resulting from this is uncertain. Therefore, the relative sizes of the three identified subgroups would probably differ depending on the study population. In the present study, it is possible that the two LBP groups were larger in our final study sample than it would have been in the targeted study sample, since the study subjects had already participated in a number of surveys on the topic of LBP and previously also received MRIs of the lumbar spine. Presumably, this study would therefore be of greater interest in relation to people with than those without LBP. This, however, does not really matter. The important issue is that these subgroups exist and that they can be easily identified through simple but distinct definitions.

Our analysis of data was explorative, using various combinations of the basic definitions “non-episode” and “pain fortnight”. Other definitions or methods of approach could of course have resulted in a different classification.

## Conclusions

It is now possible and indeed necessary to follow the 45 year old advice by LM Rowe to study LBP as a continuum [1]. In our study, frequent data collection over one year made it possible to identify three substantial subgroups in the general population; those without LBP, those with episodic LBP, and those with persistent LBP. Further subsets were identified.

### Recommendations

This was an exploratory study, meaning that replication studies are necessary to validate the findings in various study populations. It would then be better to collect data every week, as the use of blocks of two weeks may result in some misclassifications (i.e. underreporting of non-episodes).

The clinical relevance (if any) should also be investigated. Specifically the following questions arise:

•Do the various episodes in episodic LBP pain consist of distinct and singular conditions or is this subset only a lighter version of persistent pain, dormant in periods, awaiting the next opportunity to manifest itself?

•Do various subgroups, as defined on their trajectories, have different outcomes in terms of their long term natural and clinical course?

•Do these subgroups have different risk- and modifying factors?

•In other words, can the patterns of absence and presence of LBP help in the identification of specific and meaningful clinical subgroups?

## Competing interests

The authors declare that they have no competing interests.

## Authors’ contributions

PK was responsible for the original study that formed the basis for the present survey. NW and PK set up the SMS-Track study and PK was responsible for the logistics of the data collection. CLY defined the objectives and methods of the present study and carried out the analysis. Imputation of missing data was performed by NL and PK. The first draft was written by CLY and all authors read, commented upon and approved of the final manuscript.
